# The hospital costs of complications following colonic resection surgery: A retrospective cohort study

**DOI:** 10.1016/j.amsu.2020.03.013

**Published:** 2020-04-19

**Authors:** Maleck Louis, Samuel A. Johnston, Leonid Churilov, Ronald Ma, Nada Marhoon, Adele Burgess, Chris Christophi, Laurence Weinberg

**Affiliations:** a Department of Anaesthesia, Austin Health, 145 Studley Rd, Heidelberg, Victoria, 3084, Australia; b Department of Medicine, Austin Health, 145 Studley Rd, Heidelberg, Victoria, 3084, Australia; c The Melbourne Brain Centre, Royal Melbourne Hospital, 300 Grattan St, Parkville, Victoria, 3052, Australia; d Department of Finance, Austin Health, 145 Studley Rd, Heidelberg, Victoria, 3084, Australia; e Data Analytics and Research Centre, University of Melbourne, Austin Health, Heidelberg, Victoria, 3084, Australia; f Department of Surgery, University of Melbourne, Austin Health, 145 Studley Rd, Heidelberg, Victoria, 3084, Australia

**Keywords:** Colon surgery, Cost, Cost analysis, Postoperative complications

## Abstract

**Background:**

Colonic resection is a common surgical procedure associated with a high rate of postoperative complications. The aim of this observational study is to estimate the in-hospital costs of complications and to identify perioperative variables associated with complication development following colon resection surgery.

**Materials and methods:**

We conducted a single-centre cohort study with retrospective data collection of 487 patients undergoing colonic resection surgery between 2013 and 2018. Postoperative complications were graded according to the Clavien-Dindo classification system. In-hospital cost of index admission is reported in 2019 United States Dollars. Regression modelling was used to investigate the relationship of a priori selected perioperative variables and presence of complications and costs.

**Results:**

Overall complication prevalence was 69.6% (95%CI:65.5%–73.7%). Median [interquartile range] cost of patients with postoperative complications was significantly increased as compared to patients without complications ($17,963 [13,533:25,178] vs $12,578 [10,196:16,140]; p < 0.0001). Clavien-Dindo Grade I, II, III and IV complications increased costs by 15.8%, 36.8%, 169.4% and 240.1% respectively (p < 0.0001). Presence of complications was significantly associated with Charlson Comorbidity Index (Odds ratio (OR) per 1-unit increase: 1.09; 95%CI:1.02 to 1.17), preoperative albumin levels (OR per 1-unit increase: 0.94; 95%CI:0.90 to 0.98) and open as compared to laparoscopic resection (OR: 2.41; 95%CI:1.32 to 4.42).

**Conclusions:**

There is a high prevalence of complications following colonic resection surgery. Postoperative complications, including minor complications (Clavien-Dindo Grade I-II), were associated with a significant increase in hospital costs and are a key target for cost containment strategies.

## Highlights

• Almost 7 out of every 10 patients experienced one or more complications.
• Most complications were minor (Clavien-Dindo grade I-II).
• Minor complications significantly increased costs by over 20%.
• Major complications resulted in an exponential increase in costs and length of stay.
• Charlson Comorbidity Index and hypoalbuminemia are associated with complications.

## Graphical abstract

**Figure:**
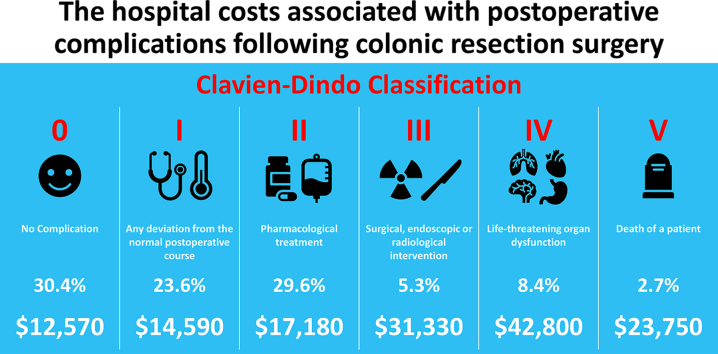

## 1 Background

### 1.1 Rationale

Cost-effective health care, particularly in the hospital setting, is crucial for the sustainability of health care systems. On the international level, health care expenditure has increased at a faster annual rate than economic growth between the years 2000 and 2016 [1]. Rising health care costs, combined with the continual necessity for high quality care, has resulted in growing demand by policymakers for high quality costing studies. In some countries, hospital expenditure is reported to represent at least one-third of total healthcare expenditure [2,3]. Therefore, it is a key target for cost containment strategies.

Vonlanthen et al. [4] reported that postoperative complications are the strongest indicators of in-hospital costs. Given that colon resection surgery is a common procedure with a high rate of postoperative adverse events relative to other major surgeries [5], it is expected to be a major contributor to hospital costs. However, there are few high-quality costing studies exploring the financial burden of complications following colon resection surgery. To address this important gap in the literature, we provide an in-depth analysis of the associations between patient factors, complications and costs following colon resection surgery. In turn, this will allow clinicians and hospital administrators to make more informed decisions about the breakdown of costs and reflect on local factors that might affect a hospital's cost of delivering care.

## 2 Objectives

The primary aim of this study was to estimate the prevalence and in-hospital costs of complications following colon resection surgery. Secondary aims included identifying perioperative variables associated with complication development and estimating the association between complications and length of stay and 30-day readmissions. We hypothesised that increased complication count and severity are associated with increased hospital costs.

## 3 Material and methods

### 3.1 Study design

We conducted a single-centre, cohort study with retrospective data collection to determine the costs associated with postoperative complications following colonic resection surgery. The Austin Health Human Research Ethics Committee approved this study and provided a waiver for participant consent (LNR/18/Austin/350). The study protocol was registered in the Australian New Zealand Clinical Trials Registry (Registration number: ACTRN12619000803190) and is accessible online from: https://clicktime.symantec.com/3W2i7J26vAXpsWcyAnTbkbv7Vc?u=http%3A%2F%2F https://www.anzctr.org.au/Trial/Registration/TrialReview.aspx?id=377549&showOriginal=true&isReview=true. There was no patient involvement in the design of this study. This manuscript is reported in accordance with STROCSS guidelines [6].

### 3.2 Setting

This study was conducted at a large, public, university teaching hospital in Australia with a high-volume colorectal service. All adult patients undergoing colonic resections between January 2013 and June 2018 were eligible for inclusion in this study. Enhanced recovery after surgery (ERAS) was implemented for all participants including systematic preoperative risk assessment and counselling and standardised perioperative management in terms of nutrition, fasting, analgesic, fluid intervention, thromboembolic prophylaxis, antimicrobial and anti-emetic regimens. Postoperative discharge criteria included full dietary intake, unassisted mobilisation, absence of surgical or medical complications and sufficient pain control.

### 3.3 Participants

Adult (>18 years of age) patients undergoing colonic resection surgery for any indication were identified using *International Statistical Classification of Diseases and Related Health Problems* 10th *Revision* (ICD-10) codes specific to colonic resection (Supplementary Table 1). Patients undergoing colonic resections of any surgical technique (open and laparoscopic) and of any urgency status (emergency and elective) were included in this study. Exclusion criteria were significant missing data preventing costing analysis, patients undergoing endoscopic mucosal, small bowel, rectal or anal resection alone and patients undergoing colonic resection which was minor and secondary to another major procedure. This was to allow the comparison of a specific homogenous patient population and focus on costs directly related to colonic resection surgeries.

### 3.4 Outcomes

Postoperative complications were defined as any deviation from the normal postoperative course during index admission and was guided by the European Perioperative Clinical Outcome definitions [7]. Severity of complications was graded according to the Clavien-Dindo classification system [8], a pre-validated classification system that categorises complication severity based on the level of treatment required: Grade I, any deviation from the normal postoperative course not requiring intervention, excluding antiemetics, antipyretics, analgesia, diuretics, electrolytes and physiotherapy; Grade II, requiring pharmacological treatment, blood transfusion or total parenteral nutrition; Grade III: requiring radiological, surgical or endoscopic intervention; Grade IV: life-threatening complication requiring intensive care management; Grade V: death [8]. Patients were stratified into groups based on the worst complication severity recorded. Length of stay was defined as the number of days from completion of surgery to discharge, excluding days on leave or in the hospital-in-the-home unit. Readmissions were defined as unplanned readmissions 30 days post discharge. Mortality was defined as death within 30 days of index admission.

Total hospital cost was defined as the sum of direct and indirect in-hospital costs of index admission for colonic resection surgery. These costs included patient care activities relating to anaesthesia, operative theatre, intensive care unit, ward, medical consults, allied health, pathology, blood products, pharmacy, radiology, medical emergency team calls and hospital-in-the-home. Costs incurred during the preoperative period were excluded from analysis to prevent potential confounding due to preoperative cost drivers. In-hospital cost of any unplanned readmissions within 30 days of discharge were added to the total cost. No patients were readmitted to another institution within 30 days of discharge. Costs were inflated to 31 March 2019 based on end of fiscal quarter Australian Consumer Price Index [9] and were then converted to United States Dollar (USD) ($) based on the market rate on 31 March 2019 [10].

Surgical technique [11,12] surgical urgency [13], Charlson Comorbidity Index (CCI) [14], preoperative anaemia [15] and preoperative albumin [[16], [17], [18], [19], [20]] were chosen a priori based on the literature for inclusion into a multivariable regression model to identify perioperative variables associated with complication development, complication count and complication severity. Complication severity was dichotomised into minor (Clavien-Dindo Grade ≤ II) and major (Clavien-Dindo Grade ≥ III) for this analysis.

### 3.5 Data sources

Data collection was performed using Cerner® electronic health records which contains prospectively recorded perioperative and patient health variables. Perioperative data collected included patient demographics, the American Society of Anesthesiologists (ASA) score [21] and the CCI [22]. Postoperative complications during index admission were coded by the Data Analytics Research and Evaluation Centre at our site and were cross-checked with complete chart review by two authors (ML and SJ) in an independent manner. In-hospital costs were calculated according to an activity-based costing methodology that allocates costs based on service volume.

### 3.6 Statistical methods

Patients with and without complications were compared using the Fisher exact and Pearson's χ^2^ tests for categorical variables and the Mann-Whitney U and Kruskal-Wallis tests for continuous variables. Multivariable logistic regression was used to investigate the relationship of a priori selected perioperative variables and presence of complications. Bootstrap quantile regression was used to estimate additional cost of complications, adjusted for surgical technique [11,12,23], surgical urgency [13,24] and preoperative anaemia [25,26] due to their potential impact on in-hospital costs following surgery identified in the literature. For each outcome, we included three quantile regression models: the 25th percentile, the 50th percentile (median), and the 75th percentile. Standard assessment of collinearity was conducted using Variance Inflation Factors (VIF) and condition numbers. Statistical software STATA/IC v.15 (StataCorp, College Station, TX, USA) and Prism 7.0 GraphPad software (La Jolla, CA, USA) were used for analysis. A p-value of ≤0.05 was considered statistically significant. No explicit correction for multiplicity of testing has been undertaken due to the exploratory nature of this study.

## 4 Results

### 4.1 Participants

497 potentially eligible patients undergoing colonic resection at our institution were identified for inclusion. 10 patients (2.0%) were excluded based on exclusion criteria specified above, with no patients excluded due to missing data. Therefore, 487 patients with a median age of 68 years [Interquartile range (IQR) 56:77] were included for analysis in this study. Patient demographics and perioperative variables for patients with and without postoperative complications are presented in Table 1.

**Table 1:** Patient demographics and perioperative variables presented as median [IQR] and count (%).

Characteristic	No complications	Complications	p-value
Number of patients	148 (30.4%)	339 (69.6%)	–
Age (years)	66 [55:75]	70 [56:77]	0.060
**Sex**			>0.999
Male	72 (48.6%)	166 (49.0%)	
Female	76 (51.4%)	173 (51.0%)	
Body mass index (Kg/m^2^)	26.8 [22.9:30.7]	26.2 [ 23.2:30.0]	0.834
**ASA**			0.0002
I	7 (4.7%)	15 (4.4%)	
II	71 (48.0%)	107 (31.6%)	
III	63 (42.6%)	174 (51.3%)	
IV	7 (4.7%)	41 (12.1%)	
V	0 (0.0%)	2 (0.6%)	
Charlson Comorbidity Index	6 [3:8]	6 [4:9]	0.084
**Principal diagnosis**			0.042
Malignant	103 (69.6%)	203 (59.9%)	
Benign	45 (30.4%)	136 (40.1%)	
Surgical urgency			0.002
Emergency	34 (23.0%)	128 (37.8%)	
Elective	114 (77.0%)	211 (62.2%)	
**Surgical technique**			<0.0001
Laparoscopic	113 (76.4%)	192 (56.6%)	
Open	22 (14.9%)	117 (34.5%)	
Laparoscopic converted to open	13 (8.8%)	30 (8.9%)	
**Surgery**			0.140
Right hemicolectomy	97 (65.5%)	204 (60.2%)	
Left hemicolectomy	10 (6.8%)	22 (6.5%)	
Total colectomy	4 (2.7%)	20 (5.9%)	
Subtotal colectomy	6 (4.1%)	32 (9.4%)	
Other	31 (20.9%)	61 (18.0%)	
Operative time (min)	221.5 [190:277]	250 [198:287]	0.022
Intensive care unit stay (hrs)	0 [0:0]	0 [0:14]	<0.0001
**Preoperative bloods**			
Albumin (g/L)	36 [33:39]	35 [30:38]	0.003
Haemoglobin (g/L)	129 [114:141]	126 [110:142]	0.246
Creatinine (μmol/L)	76 [66:94]	75 [61:93]	0.678
Bilirubin (μmol/L)	7 [4:10]	7 [4:11]	0.237
White Cell Count (x10^9^/L)	7.0 [5.9:8.8]	7.9 [6.1:10.7]	0.005
Preoperative anaemia			0.767
Yes	66 (44.6%)	157 (46.3%)	
No	82 (55.4%)	182 (53.7%)	
**Complication count**			–
0	148 (100%)	–	
1	–	107 (31.6%)	
2	–	97 (28.6%)	
3	–	54 (15.9%)	
4+	–	81 (23.9%)	
**Grade of worst complication**			–
I	–	115 (33.9%)	
II	–	144 (42.5%)	
III	–	26 (7.7%)	
IV	–	41 (12.1%)	
V	–	13 (3.8%)	
Length of stay (days)	5 [4:6]	8 [6:13]	<0.0001
30-day readmissions	17 (11.5%)	43 (12.7%)	0.766
Total cost (2019 USD)	$12,578 [10,196:16,140]	$17,963 [13,533:25,178]	<0.0001

^ASA: American Society of Anesthesiology score; USD: United States of America Dollars^.

### 4.2 Complications

Overall postoperative complication prevalence was 69.6% (339 patients; 95%CI: 65.5%–73.7%). Patients were stratified based on the most severe complication and count of complications developed (Table 1). Multivariable regression analysis of perioperative factors associated with the presence of complications, count of complications and severity of complications is demonstrated in Table 2.

**Table 2:** Perioperative variables associated with presence of complications, count of complications and complication severity.

Variable	Complication prevalence	Complication count	Complication severity
	odds ratio (95% CI; p-value)	incidence rate ratio (95% CI; p-value)	odds ratio (95% CI; p-value)
**Surgical technique**			
Laparoscopic	(reference)	(reference)	(reference)
Open	2.41 (1.32–4.42; 0.004)	1.63 (1.34–1.99; <0.0001)	4.99 (2.57–9.68; <0.0001)
Laparoscopic converted to open	1.07 (0.52–2.20; 0.850)	1.15 (0.87–1.52; 0.340)	2.69 (1.12–6.46; 0.027)
**Surgical urgency**			
Elective	(reference)	(reference)	(reference)
Emergency	1.21 (0.69–2.12; 0.504)	1.00 (0.82–1.21; 0.971)	1.21 (0.64–2.27; 0.558)
**Charlson Comorbidity Index**	1.09 (1.02–1.17; 0.011)	1.03 (1.01–1.06; 0.009)	1.07 (0.99–1.16; 0.079)
**Preoperative albumin**	0.94 (0.90–0.98; 0.005)	0.98 (0.96–0.99; 0.001)	0.93 (0.89–0.98; 0.003)
**Preoperative anaemia**			
Non-anaemic	(reference)	(reference)	(reference)
Anaemic	0.72 (0.45–1.14; 0.159)	0.98 (0.83–1.16; 0.821)	1.06 (0.60–1.89; 0.831)

Median length of stay was significantly greater in complicated patients as compared to patients without complications (Table 1). The associations between hospital length of stay and complication count and severity are demonstrated in Table 3. No significant difference was identified in 30-day readmission rates between patients with and without complications (Table 1). This relationship remained non-significant when comparing patients with major complications (Grade ≥ III) and patients without complications (p = 0.832).

**Table 3:** Median hospital costs of complications in 2019 United States Dollars and associated hospital length of stay.

	Total cost [IQR]	Total additional cost (95%CI)	P-value	Length of Stay [IQR]	P-value
Complication count					
No complications	$12,578 [10,196:16,140]	[reference]	–	5 [4:6]	–
1	$14,335 [12,029:18,009]	$1729 (365–3094)	0.013	6 [5:8]	<0.0001
2	$16,775 [13,512:22,131]	$4169 (2626–5711)	<0.0001	7 [6:11]	<0.0001
3	$18,873 [15,143:26,005]	$6375 (3928–8821)	<0.0001	10.5 [7:13.25]	<0.0001
4+	$35,040 [20,767:51,043]	$22,434 (13,584–31,284)	<0.0001	15 [9:27]	<0.0001
**Complication severity (Clavien-Dindo Classification)**					
No complications	$12,578 [10,196:16,140]	[reference]		5 [4:6]	–
I	$14,589 [11,825:18,229]	$1983 (872–3093)	<0.0001	6 [5:8]	<0.0001
II	$17,181 [13,732:21,954]	$4627 (3215–6039)	<0.0001	9 [7:12]	<0.0001
III	$31,334 [22,015:42,591]	$21,301 (12,828–29,774)	<0.0001	16 [12:27.25]	<0.0001
IV	$42,800 [26,013:67,947]	$30,194 (22,168–38,219)	<0.0001	17 [11:27]	<0.0001
V	$23,749 [16,147:38,954]	$11,143 (−1725–24,010)	0.090	6 [2.5:19.5]	0.513

Patients were stratified based on the worst grade of complication experienced and by count of complications experienced. Total additional cost was calculated using bootstrap quintile regression. Postoperative length of stay was assessed using the Mann−Whitney *U* test.

### 4.3 Cost analysis

The overall median in-hospital cost was $16,051 [IQR:12,395:22,154]. Compared to patients without complications, the additional hospital cost for patients with one or more complications was $5357 for the median patient (95%CI: 4045 to 6670; p < 0.0001), $3331 for lower costing (25th Centile) patients (95%CI: 2407 to 4254; p < 0.0001) and $9153 for higher costing (75th centile) patients (95%CI: 6567 to 11,738; p < 0.0001). Increasing complication count and increasing complication severity were significantly associated with increasing hospital costs (Table 3). The overall median cost of readmission was $5161 [IQR: 2539:11,724].

**Fig. 1:**
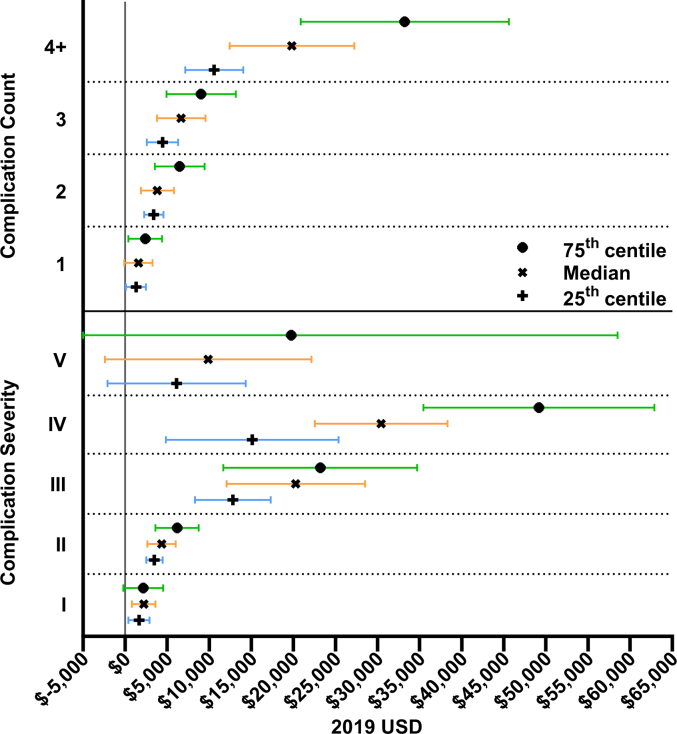
Additional cost of complication by complication severity and count in 2019 United States Dollars. Additional cost of complication for the 25th percentile, the 50th percentile (median), and the 75th percentile was calculated using multivariable bootstrap quantile regression adjusting for surgical urgency, surgical technique and preoperative anaemia; Error bars represent 95% confidence intervals.

In adjusted analysis, assuming similar surgical technique, surgical urgency and preoperative anaemia status, we estimate an additional adjusted cost of complication of $4791 for the median patient (95%CI: 3163 to 6420; p < 0.0001), $3437 for lower costing (25th Centile) patients (95%CI: 2572 to 4302; p < 0.0001) and $7504 for higher costing (75th centile) patients (95%CI: 5030 to 9978; p < 0.0001). Additional cost of complication by complication count and severity adjusted for surgical technique, surgical urgency and preoperative anaemia are demonstrated in Fig. 1.

## 5 Discussion

In a cost-analysis of postoperative complications following colonic resection surgery, we demonstrate a high complication prevalence of 69.6% with an associated significant increase in hospital costs on primary and multivariable regression analysis. Notably, minor complications (Clavien-Dindo Grade I and II) were common and significantly associated with increased costs and length of stay. Open surgery, increasing CCI and decreasing albumin levels were significantly associated with the presence of complications. These findings provide guidance for future research investigating the cost-effectiveness of targeted preventative strategies aimed at reducing postoperative complications.

Our study demonstrates a high complication prevalence following colonic resection surgery compared to previous studies [5,18,27,28]. However, there is significant variation in defining and reporting on postoperative complications in the literature which limits the ability to compare complication prevalence across studies. Many consider Grade I and II complications to be trivial and are therefore not considered when reporting the overall complication prevalence following colonic resection surgery. This is highlighted by a study conducted by de Silva et al. [28] that opted to assign patients experiencing Grade I complications to the no complication group. Furthermore, inaccurate detecting of surgical complications is a known source of bias [29,30]. This bias would be most evident in the reporting of minor complications, therefore, the comparatively high prevalence of complications in our study can be attributed to our strict adherence to the Clavien-Dindo classification system. Interestingly, the prevalence of Clavien-Dindo Grade III + complications (16.4%) in our study is in keeping with the current literature [5,18,27,28].

Our study highlights the importance of Grade I and II complications by demonstrating a high prevalence of these minor complications (53.2%). This high prevalence, combined with an associated increase in hospital costs of 15.8% and 36.8% for Grade I and II complications respectively (p < 0.0001), may translate into a considerable economic burden. In addition, patients experiencing minor complications were associated with a greater hospital length of stay when compared to patients without complications (p < 0.0001). Therefore, preventative strategies aimed at reducing the incidence of minor complications following colonic resection surgery can be expected to result in improved economic and patient outcomes.

Confirming our hypothesis, increasing complication severity was significantly associated with increasing hospital costs, with the exception of Grade V (death) complications. Grade III and IV complications were associated with an exponential increase in costs highlighting complications that require procedural intervention or Intensive Care Unit admissions as the costliest complications following colonic resection surgery. When patients are stratified based on count of complications developed, we identify that more than two thirds of patients with complications experienced multiple complications. A study by Feld et al. [31] demonstrates that the development of complications is associated with an increased relative risk of subsequent complications. As we demonstrate that increasing count of complications is associated with an exponential increase in hospital costs, this suggests that early intervention and treatment of complications has the potential to reduce the overall financial burden of complications.

Identifying perioperative variables that are associated with the development of postoperative complications enables risk stratification of patients and implementing targeted complication prevention strategies. Our study is the first to demonstrate an association between a per unit increase in CCI and postoperative complications following colonic resection surgery. Hypoalbuminemia, however, has been thoroughly investigated and identified as a strong predictor of surgical morbidity and mortality following colorectal surgery [[16], [17], [18], [19], [20]]. Our study adds to this body of literature by demonstrating a significant association between hypoalbuminemia as a continuous variable and the development of complications, reinforcing the importance of its inclusion in models that predict patient outcomes following colorectal surgery [17]. Our study did not demonstrate a significant association between preoperative anaemia and the development of complications. This is contrary to the finding of a study by Leichte et al. [15] which concluded that the presence of anaemia is associated with the development of complications in colorectal surgery. However, only severe complications, such as myocardial infarction, cerebrovascular accident and death, were considered in their study [15].

Our study did not identify a significant difference in readmission rates between patients with and without complications even when analysing patients with major complications only. Our findings were reinforced by a study by Slankamenac et al. [32] that demonstrated no significant association between complications and increased readmission rates following surgery for colorectal cancer. Merkow et al. [33] suggest that the most common indication for readmissions following surgery is complications developed post-discharge, as opposed to complications which occurred during the index admission. Therefore, research into identifying patients with increased risk of developing post-discharge complications is key to reducing readmission rates and improving clinical and economic outcomes for patients following surgery.

Our study has the following limitations. Firstly, the retrospective data collection utilised in this study subjects it to information bias, however, the impact of this bias on our study outcomes is expected to be minimal due to the extensive cross-checking required for data entry into the electronic medical records used at our institution. Secondly, although open surgery has been identified as a risk factor for complications, this finding maybe confounded by selection bias based on factors such as previous surgery or unfavourable laparoscopic conditions. Thirdly, our study was completed in a single institution which may limit our study's external validity; however, this limitation is mitigated by our centre sharing the same operative and anaesthetic protocols as other tertiary centres. Finally, our study does not investigate the long-term clinical and economic outcomes following colonic resection surgery and our cost-analysis does not consider community centred costs, which is an area for future research in this field.

## 6 Conclusions

Colonic resection surgery is associated with a high prevalence of complications which were associated with increased hospital costs and length of stay. Minor complications (Clavien-Dindo Grade I and II) were common and associated with a significant increase in costs. Increasing complication severity was also associated with increased costs. Preoperative albumin, Charlson Comorbidity Index and open surgery are associated with postoperative complications. Further research is required to identify predictors of postoperative complications to enable targeted cost-effective prevention strategies.

## Funding

This research did not receive any specific grant from funding agencies in the public, commercial, or not-for-profit sectors.

## Trial registration number

This study was registered at the Australian New Zealand Clinical Trials Registry - Registration number: ACTRN12619000803190.

## Statement of human and animal rights

The Austin Health, The Austin Health Human Research Ethics Committee approved this study and provided a waiver for participant consent - Approval no.: LNR/18/Austin/350. The authors confirm that Patients names, initials, and hospital numbers are not used.

## Provenance and peer review

Not commissioned, externally peer reviewed.

## Declaration of competing interest

The authors declare that they have no conflict of interest.
