# Supplementary material for: The hospital costs of complications following colonic resection surgery: A retrospective cohort study
Source: Ann Med Surg (Lond). 2020 Apr 19;54:37–42. doi: 10.1016/j.amsu.2020.03.013 (PMC7190696; doi:10.1016/j.amsu.2020.03.013)
Supplement: Multimedia component 1 [file mmc1.doc]

**Supplementary Material**

Supplementary Table 1: International Statistical Classification of Diseases and Related Health Problems 10th Revision (ICD-10) Codes

| **Colonic resection** | **ICD code** |
| --- | --- |
| Right hemicolectomy | 32003-01 Right hemicolectomy with anastomosis |
|  | 32005-03 Laparoscopic extended right hemicolectomy with anastomosis |
|  | 32003-03 Laparoscopic right hemicolectomy with anastomosis |
|  | 32005-01 Extended right hemicolectomy with anastomosis |
|  | 32004-01 Extended right hemicolectomy with formation of stoma |
|  | 32000-03 Laparoscopic right hemicolectomy with formation of stoma |
|  | 32004-03 Laparoscopic extended right hemicolectomy with formation of stoma |
|  | 32000-01 Right hemicolectomy with formation of stoma |
| Left hemicolectomy | 32006-00 Left hemicolectomy with anastomosis |
|  | 32006-02 Laparoscopic left hemicolectomy with anastomosis |
|  | 32006-03 Laparoscopic left hemicolectomy with formation of stoma |
|  | 32006-01 Left hemicolectomy with formation of stoma |
| Total colectomy | 32009-00 Total colectomy with ileostomy |
|  | 32012-01 Laparoscopic total colectomy with ileorectal anastomosis |
|  | 32009-01 Laparoscopic total colectomy with ileostomy |
|  | 32012-00 Total colectomy with ileorectal anastomosis |
| Subtotal colectomy | 32004-02 Laparoscopic subtotal colectomy with formation of stoma |
|  | 32005-02 Laparoscopic subtotal colectomy with anastomosis |
|  | 32005-00 Subtotal colectomy with anastomosis |
|  | 32004-00 Subtotal colectomy with formation of stoma |
| Other | 32000-02 Laparoscopic limited excision of large intestine with formation of stoma |
|  | 32003-00 Limited excision of large intestine with anastomosis |
|  | 32003-02 Laparoscopic limited excision of large intestine with anastomosis |
|  | 32000-00 Limited excision of large intestine with formation of stoma |
