# Supplementary material for: The hospital costs of complications following colonic resection surgery: A retrospective cohort study
Source: Ann Med Surg (Lond). 2020 Apr 19;54:37–42. doi: 10.1016/j.amsu.2020.03.013 (PMC7190696; doi:10.1016/j.amsu.2020.03.013)
Supplement: Multimedia component 2 [file mmc2.docx]

**Annals of Medicine and Surgery**

The following information is required for submission. Please note that failure to respond to these questions/statements will mean your submission will be returned. If you have nothing to declare in any of these categories then this should be stated.

**Please state any conflicts of interest**

All authors must disclose any financial and personal relationships with other people or organisations that could inappropriately influence (bias) their work. Examples of potential conflicts of interest include employment, consultancies, stock ownership, honoraria, paid expert testimony, patent applications/registrations, and grants or other funding.

| Declarations of interest: none |
| --- |

**Please state any sources of funding for your research**

All sources of funding should be declared as an acknowledgement at the end of the text. Authors should declare the role of study sponsors, if any, in the collection, analysis and interpretation of data; in the writing of the manuscript; and in the decision to submit the manuscript for publication. If the study sponsors had no such involvement, the authors should so state.

| This research did not receive any specific grant from funding agencies in the public, commercial, or not-for-profit sectors. |
| --- |

**Ethical Approval**

Research studies involving patients require ethical approval. Please state whether approval has been given, name the relevant ethics committee and the state the reference number for their judgement.

| The Austin Health Human Research Ethics Committee approved this study and provided a waiver for participant consent- Approval no.: LNR/18/Austin/350 |
| --- |

**Consent**

Studies on patients or volunteers require ethics committee approval and fully informed written consent which should be documented in the paper.

Authors must obtain written and signed consent to publish a case report from the patient (or, where applicable, the patient's guardian or next of kin) prior to submission. We ask Authors to confirm as part of the submission process that such consent has been obtained, and the manuscript must include a statement to this effect in a consent section at the end of the manuscript, as follows: "Written informed consent was obtained from the patient for publication of this case report and accompanying images. A copy of the written consent is available for review by the Editor-in-Chief of this journal on request”.

Patients have a right to privacy.  Patients’ and volunteers' names, initials, or hospital numbers should not be used.  Images of patients or volunteers should not be used unless the information is essential for scientific purposes and explicit permission has been given as part of the consent.  If such consent is made subject to any conditions, **the Editor in Chief** must be made aware of all such conditions.

Even where consent has been given, identifying details should be omitted if they are not essential.   If identifying characteristics are altered to protect anonymity, such as in genetic pedigrees, authors should provide assurance that alterations do not distort scientific meaning and editors should so note.

| The Austin Health Human Research Ethics Committee approved this study and provided a waiver for participant consent - Approval no.: LNR/18/Austin/350. The authors confirm that Patients names, initials, and hospital numbers are not used. |
| --- |

**Author contribution**

Please specify the contribution of each author to the paper, e.g. study concept or design, data collection, data analysis or interpretation, writing the paper, others, who have contributed in other ways should be listed as contributors.

| Louis: Study conception and design; Visualisation; Literature review; Data acquisition; Analysis and interpretation of data; Writing of manuscript – original draft, review and editing  Johnston: Study conception and design; Visualisation; Literature review; Data acquisition; Analysis and interpretation of data; Writing of manuscript – original draft, review and editing  Churilov: Study conception and design; Visualisation; Analysis and interpretation of data; Writing of manuscript – review and editing  Ma: Study conception and design; Data acquisition; Writing of manuscript – review and editing  Marhoon: Data acquisition; Writing of manuscript – review and editing  Burgess: Study conception and design; Writing of manuscript – review and editing  Christophi: Study conception and design; Writing of manuscript – review and editing  Weinberg: Study conception and design; Visualisation; Literature review; Analysis and interpretation of data; Drafting of manuscript; Writing of manuscript – original draft, review and editing  All authors have read the final manuscript and approved it for submission. |
| --- |

**Registration of Research Studies**

In accordance with the Declaration of Helsinki 2013, all research involving human participants has to be registered in a publicly accessible database.  Please enter the name of the registry and the unique identifying number (UIN) of your study.

You can register any type of research at <http://www.researchregistry.com> to obtain your UIN if you have not already registered. This is mandatory for human studies only.  Trials and certain observational research can also be registered elsewhere such as: [ClinicalTrials.gov](http://ClinicalTrials.gov) or ISRCTN or numerous other registries.

| 1. Name of the registry: Australian New Zealand Clinical Trials Registry 2. Unique Identifying number or registration ID: ACTRN12619000803190 3. Hyperlink to your specific registration (must be publicly accessible and will be checked): <https://clicktime.symantec.com/3W2i7J26vAXpsWcyAnTbkbv7Vc?u=http%3A%2F%2Fwww.ANZCTR.org.au%2FACTRN12619000803190.aspx> |
| --- |

**Guarantor**

The Guarantor is the one or more people who accept full responsibility for the work and/or the conduct of the study, had access to the data, and controlled the decision to publish

| A/Prof Laurence Weinberg  Address: Department of Anaesthesia, Austin Hospital, Heidelberg, Victoria, 3084, Australia  Email: laurence.weinberg@austin.org.au. |
| --- |
